# *Angelica Sinensis* promotes myotube hypertrophy through the PI3K/Akt/mTOR pathway

**DOI:** 10.1186/1472-6882-14-144

**Published:** 2014-05-03

**Authors:** Tzu-Shao Yeh, Cheng-Chen Hsu, Suh-Ching Yang, Mei-Chich Hsu, Jen-Fang Liu

**Affiliations:** 1 School of Nutrition and Health Sciences, Taipei Medical University, Taipei 11031, Taiwan; 2 Department of Anatomy, School of Medicine, Taipei Medical University, Taipei 11031, Taiwan; 3 Department of Sports Medicine, Kaohsiung Medical University, Kaohsiung 80708, Taiwan; 4 Graduate Institute of Sports Science, National Taiwan Sport University, Taoyuan 33301, Taiwan; 5 Department of Nutrition and Health Sciences, Chang Gung University of Science and Technology, Taoyuan 33303, Taiwan; 6 Research Center for Industry of Human Ecology, Chang Gung University of Science and Technology, Taoyuan 33303, Taiwan

**Keywords:** C2C12, Dong Quai, Muscle, IGF-1

## Abstract

**Background:**

*Angelica Sinensis* (AS), a folk medicine, has long been used in ergogenic aids for athletes, but there is little scientific evidence supporting its effects. We investigated whether AS induces hypertrophy in myotubes through the phosphatidylinositol 3-kinase (PI3K)/Akt (also termed PKB)/mammalian target of the rapamycin (mTOR) pathway.

**Methods:**

An in vitro experiment investigating the induction of hypertrophy in myotubes was conducted. To investigate whether AS promoted the hypertrophy of myotubes, an established in vitro model of myotube hypertrophy with and without AS was used and examined using microscopic images. The role of the PI3K/Akt/mTOR signaling pathway in AS-induced myotube hypertrophy was evaluated. Two inhibitors, wortmannin (an inhibitor of PI3K) and rapamycin (an inhibitor of mTOR), were used.

**Result:**

The results revealed that the myotube diameters in the AS-treated group were significantly larger than those in the untreated control group (*P* < 0.05). Wortmannin and rapamycin inhibited AS-induced hypertrophy. Furthermore, AS increased Akt and mTOR phosphorylation through the PI3K pathway and induced myotube hypertrophy.

**Conclusion:**

The results confirmed that AS induces hypertrophy in myotubes through the PI3K/Akt/mTOR pathway.

## Background

Muscle mass is a primary determinant of muscle strength, and is strongly associated with the performance of activities of daily living and the level of independence of the elderly [1-3]. The phosphatidylinositol 3-kinase (PI3K)/Akt (also termed PKB)/mammalian target of rapamycin (mTOR) pathway is recognized as a possible mechanism that regulates muscle mass [4-6]. In mammals, skeletal muscle hypertrophy occurs as a result of an increased size, instead of increased number, of preexisting skeletal muscle fibers [7,8]. The effects of this pathway on skeletal muscle are exhibited most prominently downstream of insulin-like growth factor 1 (IGF-1) signaling. The prohypertrophic activity of IGF-1 predominantly results from activation of the PI3K/Akt/mTOR signaling pathway [9]. Akt is a serine-threonine protein kinase that can inhibit the induction of muscle atrophy F box and muscle RING-finger protein 1 ubiquitin-ligases by using forkhead transcription factor FOXO1 (also called “forkhead”), resulting in the prevention of muscle atrophy [10,11]. Furthermore, activating Akt is sufficient to prevent muscle atrophy [12], and the kinase activity of Akt is essential for IGF-1-induced hypertrophy [13]. The aforementioned findings imply that the PI3K/Akt/mTOR pathway plays a pivotal role in muscle hypertrophy and atrophy.

The C2C12 cell line, a myoblast cell line derived from murine satellite cells, is used extensively as an in vitro model to study both muscle differentiation and hypertrophy [14]. The withdrawal of serum from C2C12 myoblasts leads them to exit the cell cycle and fuse into myotubes. C2C12 myotubes have been used in in vitro models to study IGF-1 mediated hypertrophic signaling pathways in skeletal muscle [9,15,16]. PI3K/Akt/mTOR activation downstream of IGF-1 can induce hypertrophy both in C2C12 cells in vitro [13] as well as in skeletal muscle in vivo [12]. Thus, C2C12 myotubes provide a useful, well-characterized, in vitro modelling system regarding the induction of hypertrophy in myotubes.

China has a long history of using natural products as ergogenic aids to enhance athletic performance. The dried root of *Angelica Sinensis* (AS) is widely used in traditional Chinese medicine to “nourish one’s vitality and enrich blood,” which means increasing the stamina of weak patients and improving their strength. The main chemical constituents of AS roots are ferulic acid, ligustilide, angelicide, brefeldin A, butylidenephthalide, butyphthalide, succinic acid, nicotinic acid, uracil, and adenine [17]. The constituents most often associated with the pharmacological activities of AS roots are ferulic acid and ligustilide (predominantly the *Z*-isomer). Ferulic acid can inhibit platelet aggregation and serotonin release, and ligustilide exhibits significant antiasthmatic and spasmolytic activities [17]. The levels of these 2 constituents are typically used as chemical markers for the quality control of AS roots [18,19]. Some of these roots are thought to exhibit proliferous properties, whereas others might exhibit myogenesis effects. Ferulic acid, an active compound derived from AS, can stimulate cell proliferation through Akt signaling [20]. Polysaccharides in AS roots can promote the proliferation and differentiation of hematopoietic stem and progenitor cells and megakaryocytic lineages through the PI3K/Akt pathway [21]. However, whether AS actually induces hypertrophy in myotubes is unknown. Therefore, we investigated whether AS induces hypertrophy in myotubes through the PI3K/Akt/mTOR pathway. An in vitro experiment regarding the induction of hypertrophy in myotubes was conducted.

## Methods

### Cell culture

Mouse skeletal muscle cells, C2C12 myoblasts, were purchased from the Bioresource Collection and Research Center (Food Industry Research and Development Institute, Hsinchu, Taiwan). Cells were maintained in 90% Dulbecco’s modified Eagle’s medium (DMEM; 11965; Gibco, Invitrogen, Carlsbad, CA, USA), supplemented with 10% fetal bovine serum (10437; Gibco, Invitrogen, Carlsbad, CA, USA) and 1% penicillin-streptomycin-amphotericin B (P/S/A; Biological industries, Kibbutz, Beit HaEmek, Israel) at 37°C in a 5% CO_2_ atmosphere. Differentiation was induced by changing the medium to DMEM containing 2% horse serum (HS) and 1% P/S/A when the cells attained 90% confluence [9,22]. The myotubes matured to striated cells by the fifth day after sowing, and the cultures were used for experiments as described previously [23]. The myotubes were assigned to 3 groups to investigate the effects of AS on myotubes: (1) non-AS supplement (normal growth medium: 2%HS/DMEM; NON); (2) IGF-1 supplement (10 ng/mL, in 2% HS/DMEM; as a positive control; IGF-1); and (3) AS supplement (10 ng/mL, in 2% HS/DMEM; AS).

### Herbal and chemical reagents

The herbal and chemical reagent stocks used were as follows: AS (10 mg/mL; HerBesta, ITS-TW, Taipei, Taiwan), wortmannin (1 μM; inhibitor of PI3K, W1628, Sigma, St. Louis, MO, USA), rapamycin (100 ng/mL; inhibitor of mTOR, R0395, Sigma, St. Louis, MO, USA), and IGF-1 (100 ng/mL, I8779, Sigma, St. Louis, MO, USA). The AS and chemicals were dissolved separately in phosphate-buffered saline ( 137 mM NaCl, 8.10 mM Na_2_HPO_4_, 2.68 mM KCl, 1.47 mM KH_2_PO_4_, pH 7.40). The stocks were stored in aliquots at -20°C. Regarding treatment, the stocks were diluted in the medium and added directly to the cultured cells according to the following final concentrations: AS (1, 10, 10^2^, 10^3^, 10^4^, 10^5^, 10^6^ ng/mL in 2% HS/DMEM), wortmannin (100 nM in DMEM), rapamycin (10 ng/mL in DMEM), and IGF-1 (10 ng/mL in 2% HS/DMEM; as a positive control for the activated PI3K/Akt/mTOR pathway).

### Assessment of *Angelica Sinensis* cytotoxicity in myotubes through an XTT assay

C2C12 cells were cultivated in a flat 96-well plate at a density of 5 × 10^3^ cells per well, and incubated for 5 d to permit the maturation of the myotubes into striated cells. AS was added to the myotubes at various concentrations (1, 10, 10^2^, 10^3^, 10^4^, 10^5^, 10^6^ ng/mL) after the myotubes matured. After 24, 48, and 72 h, an XTT (2, 3-Bis (2-methoxy-4-nitro-5-sulfophenyl)-2H-tetrazolium-5-carboxanilide inner salt) reagent (Biological industries, Kibbutz, Beit HaEmek, Israel) was added to each well according to the manufacturer’s instructions. After 2 h in the culture, cell viability was determined by measuring the absorbance at 490 nm, using a 550 BioRad plate reader (Bio-Rad, Hertfordshire, UK). Dose and time course experiments were performed in quadruplicate.

### Myotube hypertrophy based on measurement of myotube diameter

The C2C12 cells were seeded at a density of 2 × 10^5^ cells in 6-well plates (BD Biosciences, Sparks, MD, USA). The myotubes were matured after 5 d, and used in the experiments. To conduct the AS*-*induced myotube hypertrophy experiment, the myotubes were treated with AS (10 ng/mL, AS in 2% HS/DMEM) or fresh growth medium (DMEM containing 2% HS; NON) and incubated for 72 h; the myotube diameters were then determined. To determine the effects of inhibitors on AS-induced hypertrophy, the myotubes were treated with or without inhibitors (wortmannin or rapamycin) 30 min before the trials. The culture medium was replaced with IGF-1 (10 ng/mL, in 2% HS/DMEM), AS (10 ng/mL, in 2% HS/DMEM), or fresh growth medium (2% HS/DMEM; NON). The trials were conducted at 37°C in an atmosphere of 5% CO_2_. After 72 h of incubation, the myotube diameters were examined. All experiments were performed in triplicate.

The myotube diameters were determined using a light microscope (Olympus CKX41, with a 20× objective lens; Olympus, Tokyo, Japan) with a digital camera system (Olympus C7070; Olympus, Tokyo, Japan) and MediaCybernetic Image-Pro Plus software (MediaCybernetic, Bethesda, MD, USA). Each group was cultured in 3 wells, and each well was evenly divided into 9 square grid sections. Three images for each section were captured. At least 10 myotubes per image were measured. Three short-axis measurements were taken along the length of a given myotube diameter and the average was calculated.

### Western blotting

The myotubes were treated with AS (10 ng/mL) at various time points, and the time point that exhibited the highest protein expression of phosphospecific Akt and mTOR was identified using western blotting. According to the time point that exhibited the highest level phosphospecific of Akt and mTOR, the myotubes were treated with AS, and 1 μM wortmannin, an inhibitor of PI3K, was added for 30 min to break the PI3K/Akt/mTOR pathway. After incubation, the myotubes from the cell culture plate were scraped into an eppendorf tube to analyze the protein levels of phosphorylated Akt on Ser^473^ (p-Akt) and mTOR on Ser^2448^ (p-mTOR) (Cell Signaling Technology, Beverly, MA, USA). This analysis was conducted using western blotting.

Cells were lysed using a CelLytic Extraction Kit (Sigma-Aldrich, St. Louis, MO, USA) with 1% phosphatase inhibitor cocktail 3 (Sigma-Aldrich, St. Louis, MO, USA). Quantification was performed using a protein assay (Bio-Rad Laboratories, Hercules, CA, USA). Samples containing 50 μg of total protein were separated using sodium dodecyl sulfate polyacrylamide gel electrophoresis for 150 min at 120 V by applying 8% gradient gels on a Criterion electrophoresis cell (Bio-Rad Laboratories, Richmond, CA, USA).

Proteins were transferred to a polyvinylidene fluoride membrane (PALL Gelman Laboratory, Taipei, Taiwan) at a 100-mA constant current for 10 h on ice at 4°C. The membrane was blocked in a tris-buffered saline (TBS) solution containing 0.1% Tween 20 (TBS-T) and 5% nonfat dry milk for 1 h and then incubated overnight at 4°C, using commercially available rabbit polyclonal primary phosphospecific antibodies. These antibodies recognized the phosphorylated Akt on Ser^473^, mTOR on Ser^2448^ (Cell Signaling Technology, Beverly, MA, USA), and β-actin (Sigma-Aldrich, St. Louis, MO, USA).

All antibodies were diluted to a 1:500 ratio in TBS-T containing 5% nonfat dry milk (except β-actin, which was diluted to a ratio of 1:10 000). The membranes were then washed in TBS-T, incubated using a secondary antibody (horseradish peroxidase-conjugated antirabbit IgG; Sigma-Aldrich, St. Louis, MO, USA), and diluted to a ratio of 1:16 000 in TBS-T with 5% milk for 1 h, followed by washing in TBS-T. Phosphorylated proteins were visualized using enhanced chemiluminescence according to the manufacturer’s protocols (Pierce Biotechnology, Rockford, IL, USA) and quantified using MediaCybernetic Image-Pro Plus software (MediaCybernetic, Bethesda, MD, USA).

The membranes described above were incubated in Restore Western Blot Stripping Buffer (Pierce Biotechnology, Rockford, IL, USA) for 30 min and reprobed using the appropriate antibodies for detecting the total expression levels of Akt, mTOR (rabbit monoclonal primary antibody) (Cell Signaling Technology, Beverly, MA, USA), and β-actin by using western blot analysis. All experiments were performed in triplicate.

### Statistical analysis

All values were expressed as the mean ± standard deviation (SD). The myotube diameters of the 2 treatments (AS vs. NON) were compared using a Student’s *t* test. The phosphorylation levels of Akt or mTOR at various treatment time points were analyzed using a one-way analysis of variance (ANOVA). Group and treatment effect data were analyzed using a 2-way ANOVA combined with Scheffe *posthoc* analysis. Significance was determined at the *P* < 0.05 level. All tests were performed using Statistical Package for Social Science (SPSS, Chicago, IL, USA) software Version 14.0 for Microsoft Windows.

### Detection of ferulic acid in *Angelica Sinensis* by using high performance liquid chromatography

To confirm the quality of the AS, we detected its main chemical constituents. The amount of ferulic acid in the AS was analyzed using a high performance liquid chromatographic method [24]. Quantification was accomplished using a comparison of the peak areas of the sample with those of the reference standard. The amount of ferulic acid in the AS was 0.61 mg/g (Additional file 1: Figure S1 and S2).

## Results

### Concentration and time effects of *Angelica Sinensis* on the viability of myotubes

The viability of cells in the group without AS treatment was expressed as 100%. As shown in Table 1, at 24 h, the cell viability of myotubes decreased by 9%, 16%, and 26% when exposed to 10^4^, 10^5^, and 10^6^ ng/mL of AS, respectively, compared with the cells in the untreated control group. At 48 h, the cell viability of the myotubes decreased by 9%, 25%, and 31% when exposed to 10^4^, 10^5^, and 10^6^ ng/mL of AS, respectively, compared with the cells in the untreated control group. At 72 h, the cell viability of the myotubes decreased by 9%, 25%, and 32% when exposed to 10^4^, 10^5^, and 10^6^ ng/mL of AS, respectively, compared with the cells in the untreated control group. The cell viability at concentrations of 10^5^ and 10^6^ ng/mL of AS was significantly decreased compared with the control group after the same period of culturing (*P* < 0.05). The results indicated that AS was not harmful to myotubes at concentrations of 1, 10, or 10^2^ ng/mL. Therefore, an AS concentration of 10 ng/mL was used to induce hypertrophy in the experiment.

**Table 1:** **Effect of *Angelica Sinensis *** **on viability of myotubes (%)**

		**Concentration *of Angelica Sinensis *** **treatment (ng/mL)**						
	**Control**	**1**	**10**	**10**^**2**^	**10**^**3**^	**10**^**4**^	**10**^**5**^	**10**^**6**^
**24 h**	100.00 ± 3.53	99.45 ± 4.09	99.89 ± 5.64	98.18 ± 4.52	93.94 ± 6.59	91.24 ± 4.34	83.62 ± 5.07*	74.41 ± 5.88*
**48 h**	100.00 ± 3.42	100.15 ± 5.34	100.04 ± 5.56	100.13 ± 5.75	93.49 ± 4.44	91.22 ± 4.09	75.08 ± 5.15*	68.82 ± 4.65*
**72 h**	100.00 ± 3.75	98.56 ± 3.49	99.42 ± 5.21	99.17 ± 4.86	91.31 ± 3.92	90.64 ± 3.59	74.93 ± 5.26*	68.45 ± 4.53*

Values are mean ± SD (n = 4). Data were analyzed with one-way ANOVA with dose factors. *Significantly different compared with the control at the same time of culture (Scheffe’s *post hoc* analysis, *P* < 0.05).

### Myotube hypertrophy induced by *Angelica Sinensis*

**Figure 1:**
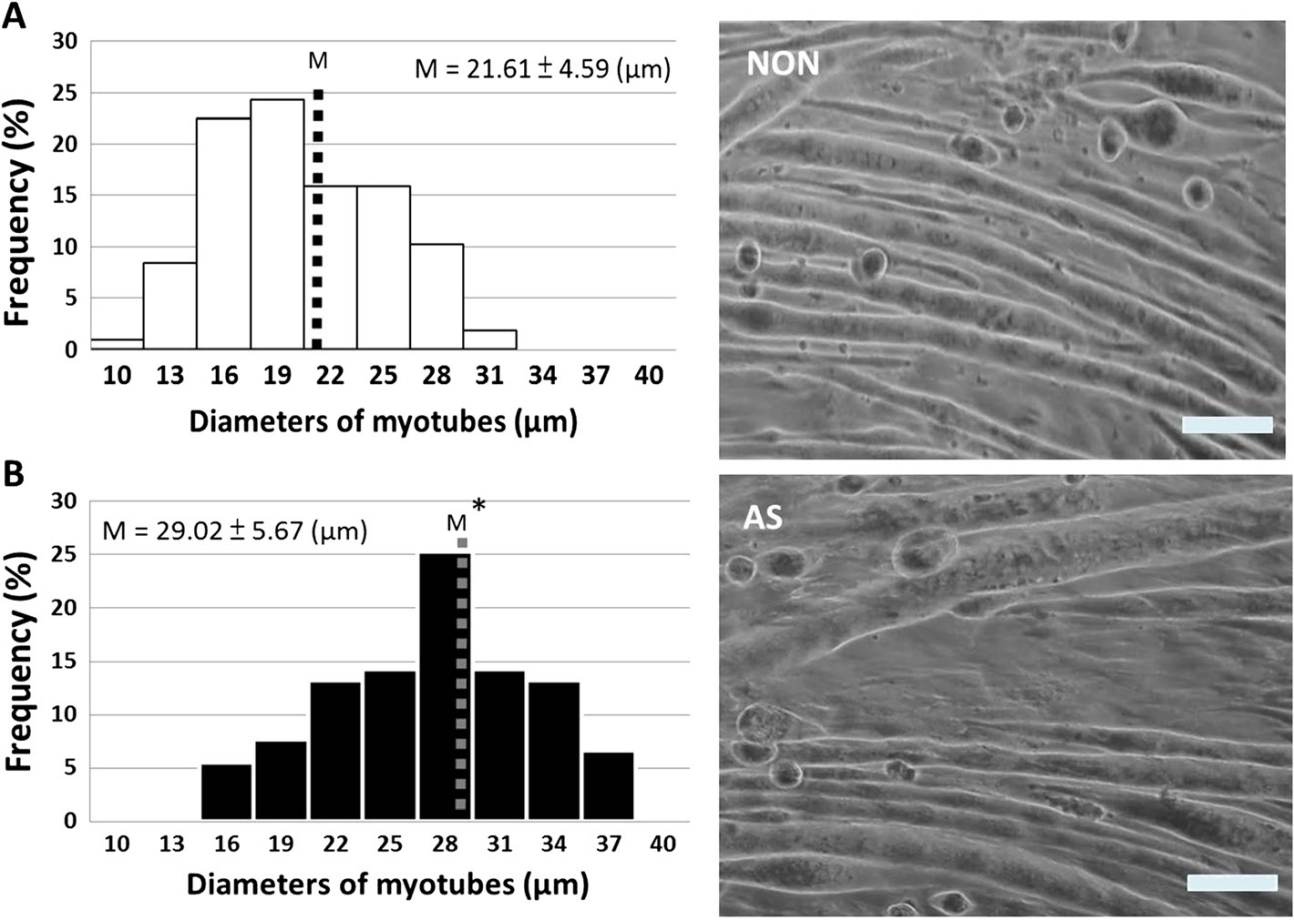
***Angelica Sinensis *(AS) induced myotube hypertrophy after 72 h treatment.** Histograms of myotube diameters derived from microscope images. Right panel shows a representative myotubes of image. Scale bar = 50 μm. Quantification of mean myotube diameter (M) ± SD. **(A)** NON, non- *Angelica Sinensis* supplements, Dulbecco’s modified Eagle’s medium (DMEM) containing 2% horse serum (HS), n = 107; **(B)** AS, 10 ng/mL of AS in 2% HS/DMEM, n = 91; n-value represented the myotube numbers from image. The mean of myotube diameters (M) was significantly enlarged in AS group. Data were analyzed with the Student’s *t*-test. Statistical significance: *P < 0.05 vs. NON.

To determine whether AS is functionally critical for myotube hypertrophy, the influence of AS (10 ng/mL) on myotube thickness was examined. After 72 h of incubation, highly thickened myotubes were observed in the AS-treated group. The myotube diameter of 2 groups (NON and AS) exhibited normal distribution. The result indicated that the average myotube diameter in the AS group increased 1.34 ± 0.13 fold compared with the NON group (*P* < 0.05, Figure 1). This clearly revealed that AS induced myotube hypertrophy.

### Involvement of the PI3K/Akt/mTOR pathway in *Angelica Sinensis*-induced myotube hypertrophy

**Figure 2:**
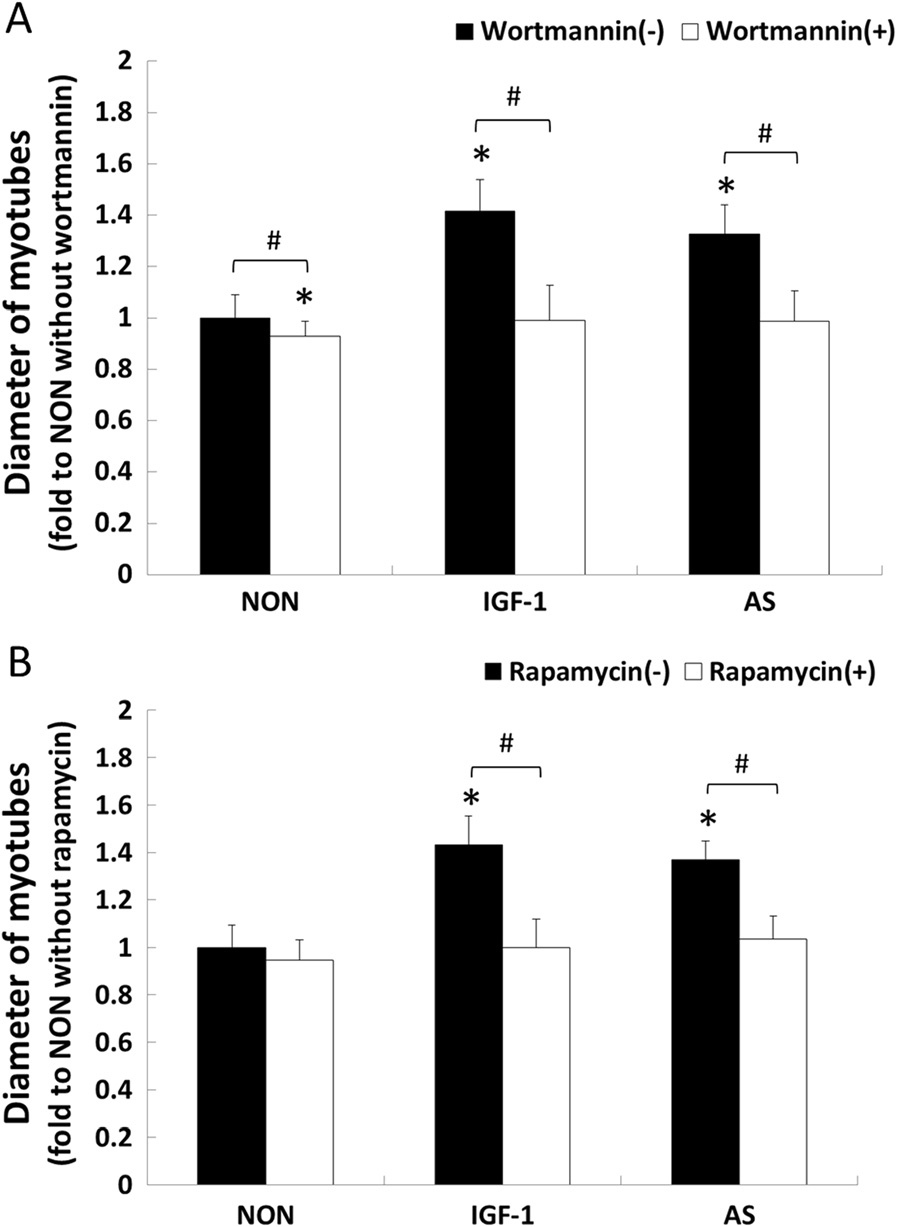
**Effects of inhibitors on *Angelica Sinensis *(AS)-induced hypertrophy after 72 h treatment.** C2C12 myotubes were treated for 72 h with: 100 nM wortmannin in DMEM, an inhibitor of PI3K **(A)**; 10 ng/mL rapamycin in DMEM, an inhibitor of mTOR **(B)**. The vertical axes indicated relative myotube diameter normalized to the mean diameter of non-AS supplements (error bars: SD). Black bars represented myotubes without inhibitors, and white bars were myotubes with inhibitors, respectively. NON, non-AS supplements, 2% HS/DMEM; IGF-1, Insulin growth factor 1 stimulation, 10 ng/mL of IGF-1 in 2% HS/DMEM; AS, *Angelica Sinensis* treatment, 10 ng/mL of AS in 2% HS/DMEM. (NON, n = 103; IGF-1, n = 92; AS, n = 91; n-value represented the myotube numbers from image). Data were analyzed with two-way ANOVA. *Significantly different compared with the NON without inhibitor: wortmannin **(A)** and rapamycin **(B)** (Scheffe’s *post hoc* analysis, *P* < 0.05). #Significant inhibitor effect in the same group (Scheffe’s *post hoc* analysis, *P* < 0.05).

To examine the role of the PI3K/Akt/mTOR signaling pathway in AS-induced myotube hypertrophy, pharmacologic experiments were conducted using inhibitors that interfered with this pathway. IGF-1 stimulation that activated the pathway was used as a positive control. The PI3K inhibitor, wortmannin, reduced the diameters of AS-treated myotubes by 25% (*P* < 0.05, Figure 2A, AS), and the diameters of the positive controls by approximately 30% (*P* < 0.05, Figure 2A, IGF-1). The mTOR inhibitor, rapamycin, behaved similarly to wortmannin (Figure 2B). These results indicated that the PI3K/Akt/mTOR pathway played a crucial role in AS-induced myotube hypertrophy.

### Akt phosphorylation induced by *Angelica Sinensis*

**Figure 3:**
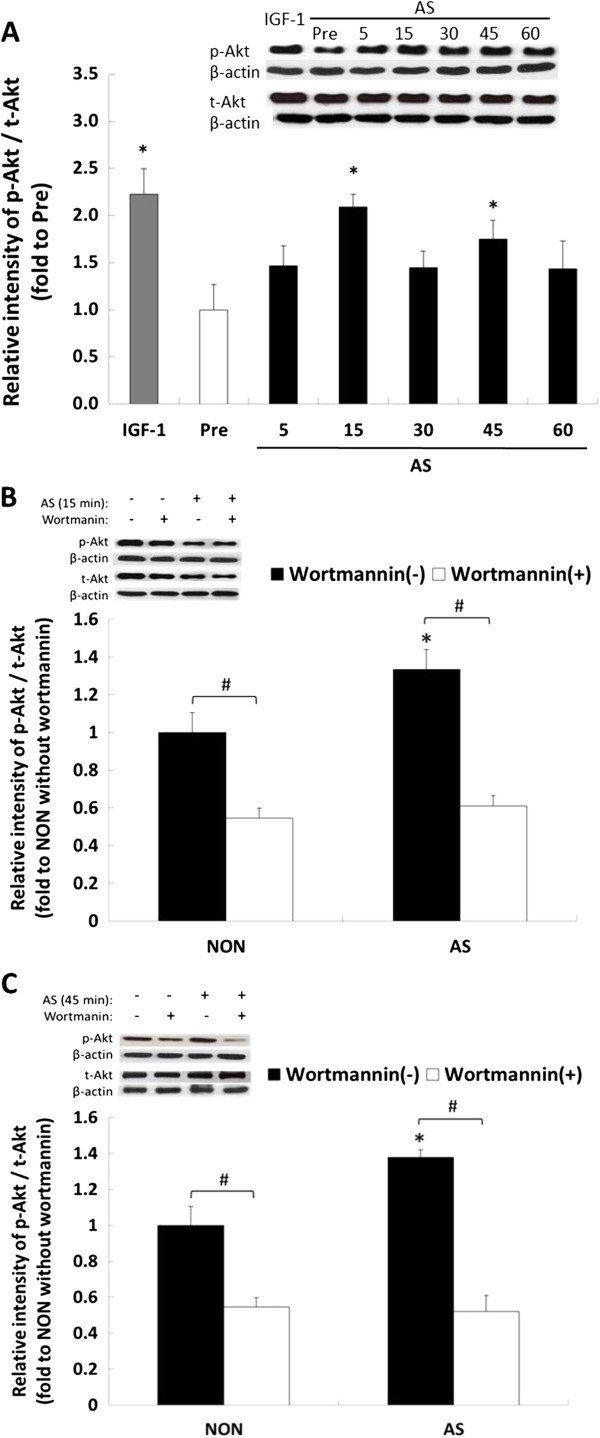
**Phosphorylation of Akt induced by *Angelica Sinensis *(AS). (A)** Upper panel showed a representative result of western blot analysis of total- Akt (t-Akt) and phosphor-Akt (p-Akt) levels in the myotubes treated with 10 ng/mL of IGF-1 in 2% HS/DMEM for 45 min, or AS (10 ng/mL of AS in 2% HS/DMEM) for 5 to 60 min. **(B)** Akt phosphorylation level at 15 min in wortmannin-treated myotubes. **(C)** Akt phosphorylation level at 45 min in wortmannin-treated myotubes. p-Akt and t-Akt were normalized by individual β-actin. The results of the densitometric analysis of the western blot membranes [upper panels in **(A)**, **(B)** and **(C)**] were depicted in the lower panels as the ratio of p-Akt against the t-Akt signal (mean ± SD, n = 3), respectively. Vertical axis represented relative p-Akt level compared with pre-treated myotubes **(A)**, or non-treated myotubes **(B)** and **(C)**. Data were analyzed with one-way ANOVA with time factors in (A). Data were analyzed with two-way ANOVA with group and inhibitor treat as factors in **(B)** and **(C)**. *Significant time effect compared with pre-treat in **(A)** (Scheffe’s *post hoc* analysis, *P* < 0.05). *Significantly different compared with the NON without inhibitor wortmannin in **(B)** and **(C)** (Scheffe’s *post hoc* analysis, *P* < 0.05). #Significant inhibitor effect in the same group (Scheffe’s *post hoc* analysis, *P* < 0.05).

Following the aforementioned indication of the role of the PI3K/Akt/mTOR pathway in AS-induced myotube hypertrophy, we investigated whether Akt phosphorylation was promoted by AS. First, a time-course analysis was performed using western blotting, which showed that 15 and 45 min of AS treatment significantly elevated the Akt phosphorylation level (*P* < 0.05), as did IGF-1 stimulation regarding the positive control (*P* < 0.05, Figure 3A). Second, further investigation showed that Akt phosphorylation induced by 15 min of AS treatment was significantly reduced beyond the non-AS supplement’s level (*P* < 0.05, Figure 3B), using wortmannin; essentially the same results were obtained in the samples regarding Akt phosphorylation induced by 45 min of AS treatment (Figure 3C). These data suggested that AS promoted Akt phosphorylation through the PI3K pathway, which was observed in the case of IGF-1 stimulation.

### Mamallian target of rapamycin phosphorylation induced by *Angelica Sinensis*

**Figure 4:**
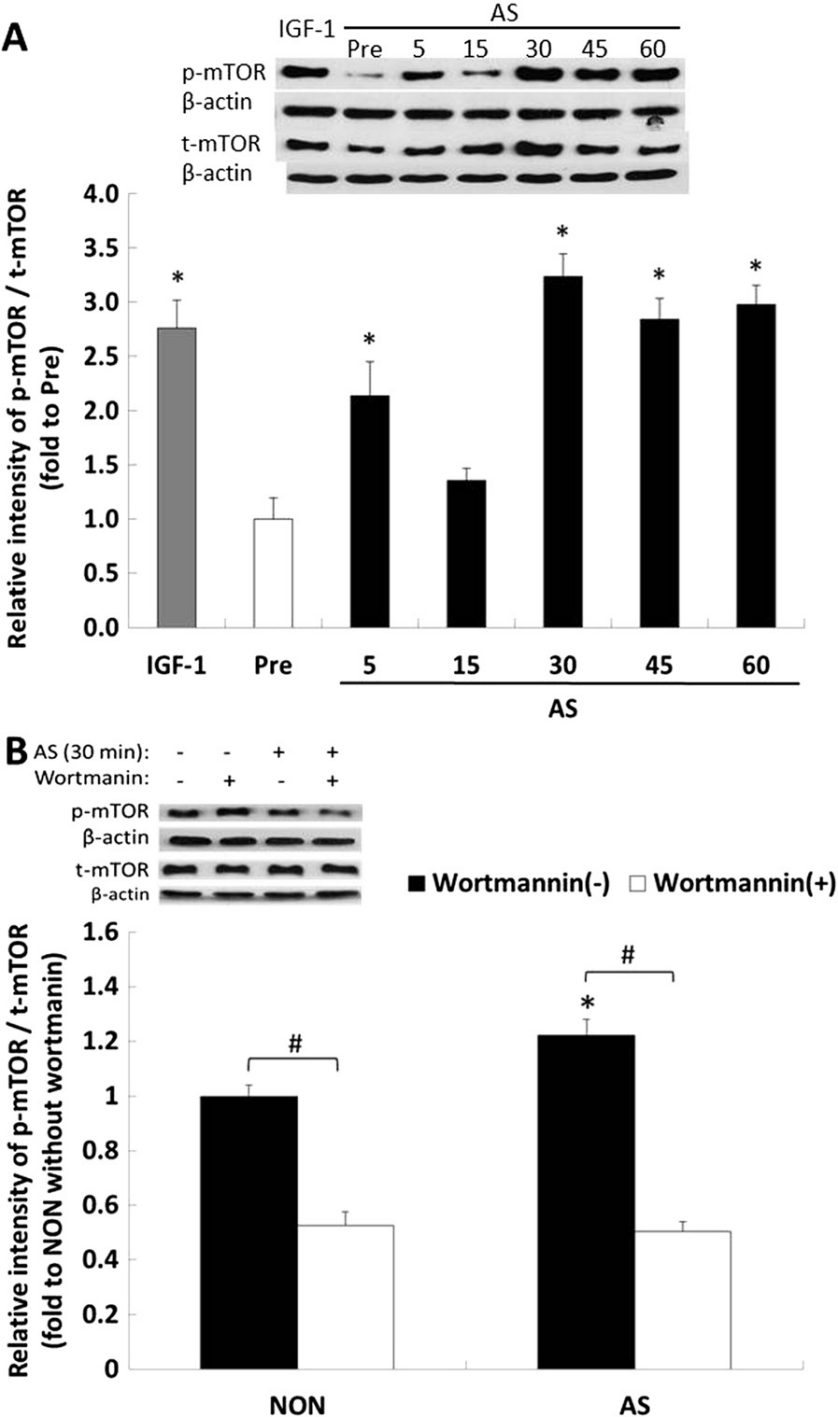
**Phosphorylation of mTOR induced by *Angelica Sinensis *(AS). (A)** Upper panel showed a representative result of western blot analysis of total (t-mTOR) and phosphor-mTOR (p-mTOR) levels in the myotubes treated with 10 ng/mL of IGF-1 in 2% HS/DMEM for 45 min, or AS (10 ng/mL of AS in 2% HS/DMEM) for 5 to 60 min. **(B)** mTOR phosphorylation level at 30 min in wortmannin-treated myotubes. p-mTOR and t-mTOR were normalized by individual β-actin. The results of the densitometric analysis of the western blot membranes [upper panels in **(A)** and **(B)**] were depicted in the lower panels as the ratio of p-mTOR against the t-mTOR signal (mean ± SD, n = 3), respectively. Vertical axis represented relative p-mTOR level compared with pre-treated myotubes (A), or non-treated myotubes **(B)**. Data were analyzed with one-way ANOVA with time factors in **(A)**. Data were analyzed with two-way ANOVA with group and inhibitor treat as factors in **(B)**. *Significant time effect compared with pre-treat in **(A)** (Scheffe’s *post hoc* analysis, *P* < 0.05). *Significantly different compared with the NON without inhibitor wortmannin in **(B)** (Scheffe’s *post hoc* analysis, *P* < 0.05). #Significant inhibitor effect in the same group (Scheffe’s *post hoc* analysis, *P* < 0.05).

The procedure for this experiment resembled the aforementioned time-course analysis. Results showed that 30 min of AS treatment significantly elevated the mTOR phosphorylation level (*P* < 0.05), as did IGF-1 stimulation in the positive control group (*P* < 0.05, Figure 4A). However, a decrease was observed in the phosphorylation level of mTOR between 5 and 30 min after the AS treatment of the myotubes. The mTOR phosphorylation behaved similarly to Akt phosphorylation, but more powerfully expressed the hypertrophy signal in the AS-treated sample. Furthermore, the elevated phosphorylation induced using AS treatment for 30 min was significantly reduced using wortmannin compared with the non-AS supplemented group (*P* < 0.05, Figure 4B). The non-AS supplemented group exhibited a similar reaction. Additionally, the wortmannin inhibition of phosphorylation levels was not significantly different between the 2 groups. As shown in Figures 3B and 4B, the AS-induced hypertrophy through the PI3K/Akt/mTOR phosphorylation pathway was completely inhibited using wortmannin; however, it was unclear whether the hypertrophy was solely induced by the PI3K/Akt/mTOR pathway. Nonetheless, after wortmannin inhibition the AS-induced phosphorylation was significantly reduced. Therefore, PI3K undoubtedly played a major role in hypertrophy.

## Discussion

The primary finding of the present study was that AS increased myotube hypertrophy through the PI3K/Akt/mTOR pathway. According to a thorough review of relevant research, this is the first study to demonstrate that myotube hypertrophy induced by AS treatment occurs through the PI3K/Akt/mTOR pathway, as does IGF-1-induced hypertrophy. Furthermore, treatment with AS increases the activation of the PI3K/Akt/mTOR pathway.

The PI3K/Akt/mTOR pathway was investigated to understand the mechanism through which AS promotes hypertrophy. Activating this pathway promotes skeletal muscle hypertrophy and prevents muscle atrophy [25] because the kinase activity of Akt is essential for IGF-1-induced hypertrophy [13]. Akt is a serine-threonine protein kinase that can induce protein synthesis and block the transcriptional upregulation of key mediators of skeletal muscle atrophy [26]. No previous studies that examined AS have investigated the regulation of the PI3K/Akt/mTOR pathway in myotube hypertrophy. Immunoblotting by using antibodies against activated or total Akt and mTOR revealed that AS activated this pathway in myotubes, which clarified AS’s hypertrophic effects on myotubes.

Wortmannin, a specific inhibitor of PI3K, was used to distinguish whether AS activated Akt through the classical PI3K pathway or through an alternative pathway. Wortmannin attenuated Akt activation was induced using AS, which demonstrated that Akt activation by using AS is dependent on the PI3K pathway. In this study, we observed that wortmannin inhibited hypertrophy that was promoted using AS, confirming that PI3K-mediated Akt activation by using AS was necessary to induce hypertrophy in myotubes.

Rapamycin is a pharmacologic agent that binds to mTOR and inhibits its functioning [27]. In vitro, when applied to myotube cultures, rapamycin blocks activation of p70S6K downstream of either activated Akt or IGF-1 stimulation [27,28]. In this study, we observed that rapamycin inhibited the hypertrophy promoted using AS, which confirmed that Akt-mediated mTOR activation by using AS is necessary to induce hypertrophy in myotubes.

As shown in Figure 4, we observed that myotubes treated with AS for 15 min or longer had significantly increased levels of PI3K-mediated Akt activation on Ser^473^ (*P* < 0.05) resulting in hypertrophy, and that activating Akt and its resulting downstream effects was triggered by treatment with AS. AS is responsible for the increase in the activation of mTOR phosphorylation at Ser^2448^ observed 30 min after AS treatment (Figure 4A).

Akt is a serine-threonine kinase involved in the regulation of cellular metabolism and has been shown to induce rapid skeletal muscle hypertrophy in vivo [29]. Phosphorylation of Ser^473^ is required for maximal activation of Akt and it appears that Akt might have a relatively short activation period after nutritional stimulation is activated by protein growth factors [30-32]. In this study, the protein level of Akt phosphorylation was observed as early as 5 min after AS treatment and reached maximum protein expression at 15 min. These results were consistent with previous reports [30].

This study revealed that AS increased myotube diameter and seemed to be mediated via the mTOR pathway. Because 2% horse serum was used in all treatment media throughout the study, the mechanism might have resulted from the direct effect of AS on the mTOR pathway or the enhanced mTOR pathway caused by facilitation of the binding of IGF-1 to its receptor. However, our results revealed that myotube diameter in the AS group was significantly thickened compared with that of the NON group, but not the IGF-1 group (Figure 2). According to our in vitro data, even if horse serum contained IGF-1, AS-induced myotube hypertrophy did not entirely enhance the mTOR pathway by facilitating the binding of IGF-1 to its receptor (Figure 2). We suggest that further study by using a serum free medium is required to investigate how AS activates the PI3K/Akt/mTOR pathway.

mTOR is a 289 kDa serine-threonine kinase partially downstream of Akt and is responsible for the complex integration of anabolic stimuli mediating cell growth [32]. Although AKT phosphorylated mTOR at 2 COOH-terminal sites (Thr^2446^ and Ser^2448^) in vitro, Ser^2448^ was the major phosphorylation site in insulin-stimulated or -activated AKT-phosphorylating human skeletal muscle cells [32,33]. Phosphorylation of mTOR at Ser^2448^ has become a popular biomarker for the activation state of skeletal muscle hypertrophy signaling pathways and the activation status of mTOR [28,30,34]. A previous study reported that mTOR is a direct substrate for the Akt kinase and identified Ser^2448^ as the Akt target site in mTOR [35]. In addition to the regulation of mTOR by using the PI3K/Akt pathway, others have provided evidence that the Ser^2448^ phosphorylation primarily reflects a feedback signal to mTOR from its downstream target, p70S6 kinase (S6K1) [36]. Figure 4 shows that 30 min of AS treatment significantly elevated the mTOR phosphorylation level at Ser^2448^. The negative regulation of skeletal muscle hypertrophy through the p70S6 pathway was a possible reason for the increased phosphorylated mTOR at the Ser^2448^ site between 30 and 60 min that was observed. However, downstream signaling factors were required to sustain AKT/mTOR signaling. Our results suggested that, at least regarding the cell types examined in this study, Ser^2448^ phosphorylation exhibited both direct and indirect reactions by using AS stimulation and markers of Akt activation.

## Conclusion

The results confirm that AS induces hypertrophy in myotubes through the PI3K/Akt/mTOR pathway.

## Competing interests

The authors declare that they have no competing interests.

## Authors’ contributions

TSY designed the experiments, performed the laboratory experiments, analyzed the data, interpreted the results, prepared figures, and wrote the manuscript. CCH and SCY edited and revised manuscript. MCH and JFL supervised the study design and revised the manuscript. All authors discussed the results and implications and commented on the manuscript at all stages. All authors read and approved the final manuscript.

## Pre-publication history

The pre-publication history for this paper can be accessed here:

http://www.biomedcentral.com/1472-6882/14/144/prepub

## Supplementary Material

Additional file 1: Figure S1 The chromatogram of ferulic acid (FA) in *Angelica Sinensis*. **Figure S2.** Various concentration of *Angelica Sinensis* (AS) induced myotube hypertrophy after 72 h treatment. Scale bar = 50 μm. Click here for file
