# Supplementary material for: Angelica Sinensis promotes myotube hypertrophy through the PI3K/Akt/mTOR pathway
Source: BMC Complement Altern Med. 2014 May 3;14:144. doi: 10.1186/1472-6882-14-144 (PMC4229743; doi:10.1186/1472-6882-14-144)
Supplement: Additional file 1: Figure S1 — The chromatogram of ferulic acid (FA) in Angelica Sinensis. Figure S2. Various concentration of Angelica Sinensis (AS) induced myotube hypertrophy after 72 h treatment. Scale bar = 50 μm. [file 1472-6882-14-144-S1.docx]

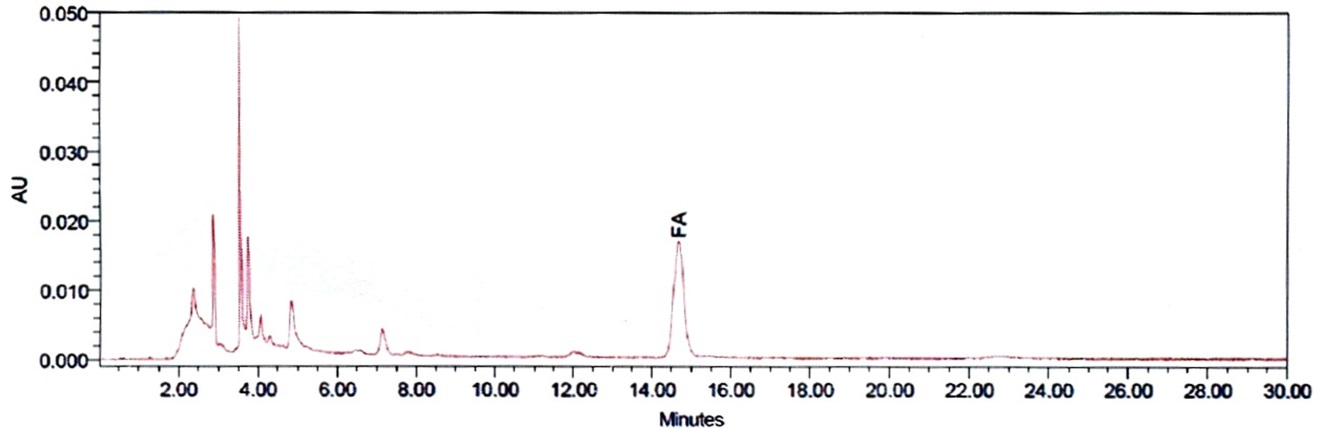


**Figure S1. The chromatogram of ferulic acid (FA) in *Angelica sinensis*.**

**
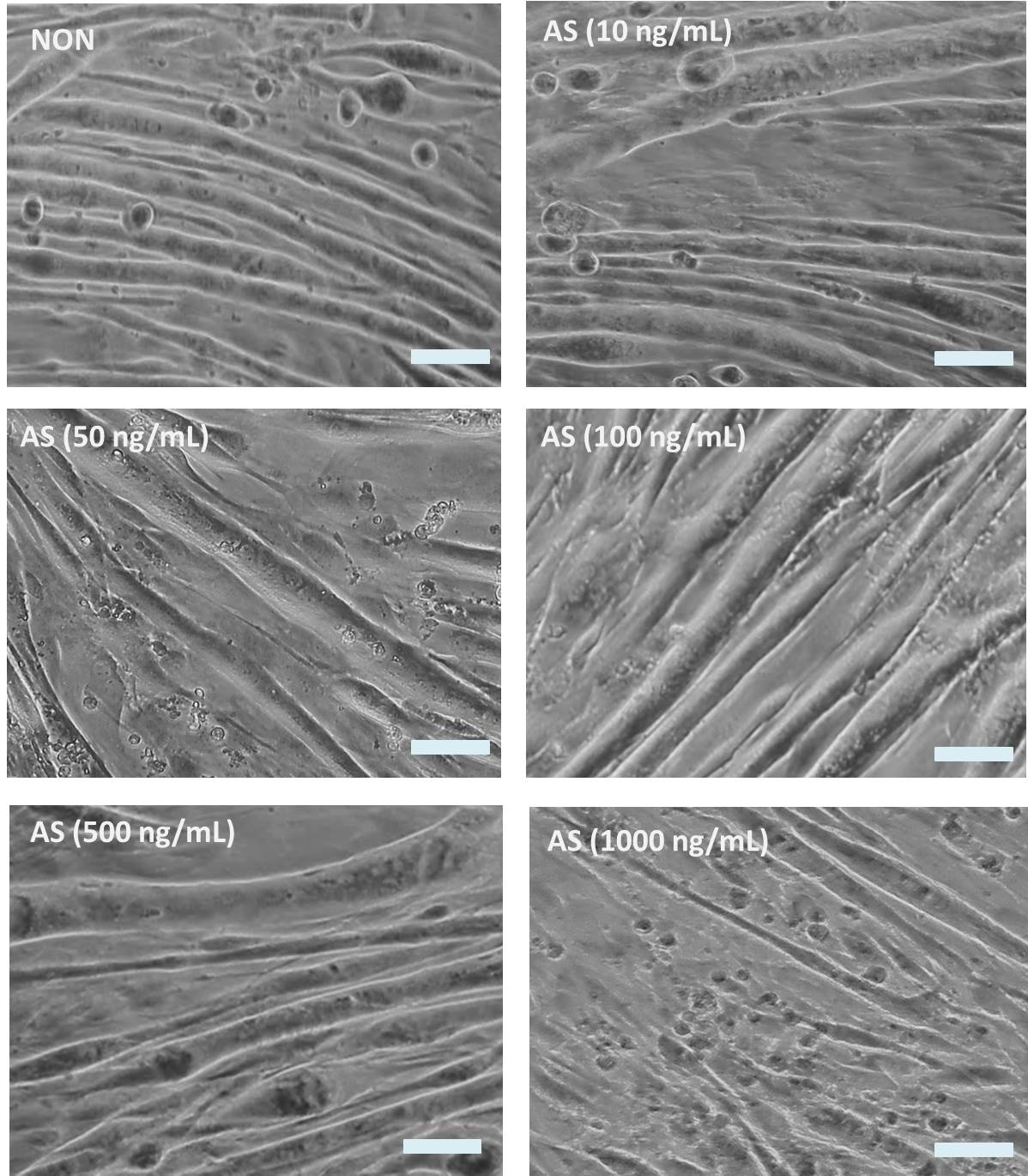
**

**Figure S2.** **Various concentration of *Angelica sinensis* (AS) induced myotube hypertrophy after 72 h treatment.** Scale bar = 50 μm.
